# Emergence of New Insect-Restrictive Viruses in the Amazon Region

**DOI:** 10.1128/genomeA.00131-15

**Published:** 2015-03-19

**Authors:** Márcio R. T. Nunes, Sandro P. Silva, Valéria L. Carvalho, Janaina M. Vasconcelos, Daisy E. A. Da Silva, Layanna F. Oliveira, Joaquim P. Nunes Neto, Sueli G. Rodrigues, Raimunda S. S. Azevedo, Hamilton A. O. Monteiro, Jedson F. Cardoso, Hilda Guzman, Robert Tesh, Pedro F. C. Vasconcelos, João L. S. G. Vianez-Júnior, Lívia C. Martins

**Affiliations:** a Department of Arbovirology and Hemorrhagic Fevers, Evandro Chagas Institute, Ministry of Health, Ananindeua, Brazil; b Center for Technological Innovation, Evandro Chagas Institute, Ministry of Health, Ananindeua, Brazil; c Department of Pathology, Center for Biodefense and Emerging Infectious Diseases, University of Texas Medical Branch, Galveston, Texas, USA

## Abstract

The complete genome was determined for 12 viruses isolated from 8 different pools of mosquitoes (*Culex* sp. and *Psorophora ferox*) collected at Brejeira farm, Canaan dos Carajas, Para state in northern Brazil. Eight of the viruses were distantly related to Piura virus, hereafter designated as Brejeira virus; the other 4 were similar to Wallerfield virus.

## GENOME ANNOUNCEMENT

During the past decade, a growing number of novel insect-specific viruses have been detected in naturally infected mosquitoes (1). These insect-specific or insect-restricted viruses belong to viral families containing arboviruses as well as completely new taxa, demonstrating the great diversity of viruses that are present in mosquitoes (2).

One of these new taxa of insect-specific viruses is the recently proposed new taxon *Negevirus*. Negeviruses were isolated from mosquitoes and phlebotomine sand flies collected in North and South America, Africa, and Asia (1, 3, 4).

All isolates used in this study were collected during an ecoepidemiological study carried out in the municipality of Canaã dos Carajás (6°31′56.73″S, 49°51′4.47″W), State of Pará, Northern Brazil, and represent low-passage viruses in C6/36 cells. Viruses were provided by the Department of Arbovirology and Hemorrhagic Fevers, Pan American Health Organization, World Health Organization Collaborating Center for Arbovirus Research.

Mosquito pools were separated by species (1–30 mosquitoes), kept in 2-mL Eppendorf tubes containing D-PBS and one 3-mm metal bead (Tungsten Carbide Beads), and macerated using the TissueLyser II (Qiagen) at 25 Hz for 1 min. Mosquito macerates were centrifuged at 3,000 rpm, and the supernatant was used for inoculation in the C6/36 cells, in an attempt to isolate the virus. The supernatant of infected C6/36 cells was harvested after 75% to 90% cytopathic effect production and used for RNA extraction.

RNA was extracted using the QIAamp Viral RNA minikit. Full-length genomes were obtained using the Ion Torrent PGM (Life Technologies), as previously described (5). The sequencing steps were carried out at the Genomic Core of the Center for Technological Innovation, Evandro Chagas Institute.

All genomes were assembled and compared to viral sequences in the NCBI database using BLASTx, and 12 Negevirus-like viruses were identified. Maximum pairwise nucleotide identities between four Wallerfield viruses (this study), showed 97.4% to 97.2% on the nucleotide level with a Wallerfield virus (NC023440) isolated in Trinidad and Tobago, suggesting the detection of four isolates of one novel strain from Brazil.

The Brejeira virus (a new virus from the Amazon region) was mostly related to Piura virus (JQ675607), with maximum pairwise nucleotide identities between 68.0% and 67.2% on the nucleotide level, suggesting the detection of eight isolates of one novel Negevirus-like species identified in Brazil.

Similar to other Negevirus-like sequences, four domains were predicted for the first open reading frame (ORF1), a methyltransferase domain, an rRNA methyltransferase domain, a helicase domain, and an RdRp domain. No conserved domains were identified for ORF2 and ORF3.

The biological and potential public health importance of the Negeviruses has yet to be determined, but some possible scenarios and areas of future research are outlined below. In addition to their broad geographic distribution, the Negeviruses appear to infect a wide range of hematophagous insects (mosquitoes of the genera Culex, Aedes, and Anopheles, as well as sand flies of the genus Lutzomyia) (1).

In this scenario, it is important to assess how these viruses interact with the environment and contribute or discourage the transmission of other arboviruses of interest to human and/or animal health.

### Nucleotide sequence accession numbers.

The complete sequences have been deposited in GenBank under the accession numbers KM350504 through KM350507 (four different Wallerfield viruses) and KM350508 through KM350515 (eight different Brejeira viruses).
